# Chronic Cortisol Exposure Reshapes Chromatin Accessibility and Gene Expression: Implications for Growth and Welfare in Rainbow Trout (*Oncorhynchus mykiss*)

**DOI:** 10.3390/ijms27198482

**Published:** 2026-09-23

**Authors:** Leonidas Larrondo, Cristian Reyes, Katalina Llanos-Azócar, Rodrigo Zuloaga, Daniela Aravena-Canales, Camila Godoy-Diaz, Alfredo Molina, Giorgia Daniela Ugarte, Juan Antonio Valdés

**Affiliations:** 1 Laboratorio de Biotecnología Molecular, Facultad de Ciencias de la Vida, Universidad Andres Bello, Santiago 8370186, Chile; l.larrondocazanga@uandresbello.edu (L.L.); c.reyescampos@uandresbello.edu (C.R.); k.llanosazcar@uandresbello.edu (K.L.-A.); rodrigo.zuloaga.r@gmail.com (R.Z.); daniela.aravena.canales@gmail.com (D.A.-C.); c.godoydiaz@uandresbello.edu (C.G.-D.); amolina@unab.cl (A.M.); giorgia.ugarte@unab.cl (G.D.U.); 2 Interdisciplinary Center for Aquaculture Research–Applied Research (INCAR^2^), Universidad Andres Bello, Santiago 8370186, Chile; 3 Centro de Investigacion Marina Quintay (CIMARQ), Universidad Andres Bello, Quintay 2340000, Chile

**Keywords:** cortisol, chronic stress, rainbow trout, ATAC-seq, RNA-seq, welfare

## Abstract

Cortisol, the principal glucocorticoid in teleosts, coordinates the physiological response to stress by regulating metabolism, protein turnover, and growth. Although the molecular effects of acute cortisol exposure have been extensively characterized, the epigenomic mechanisms underlying skeletal muscle remodeling during prolonged cortisol elevation remain poorly understood. Here, we investigated how chronic cortisol reshapes chromatin accessibility and gene expression in rainbow trout (*Oncorhynchus mykiss*) skeletal muscle using an integrative ATAC-seq and RNA-seq approach. Fish received a single intraperitoneal injection of cortisol (50 μg g^−1^ body weight) formulated in coconut oil for sustained hormone release and were sampled after 14 days. Cortisol treatment significantly increased plasma cortisol levels, reduced specific growth rate, and increased skeletal muscle protein carbonylation, indicating impaired growth and oxidative protein damage. ATAC-seq revealed extensive chromatin remodeling, including 126,780 cortisol-specific accessible regions and 3393 genes associated with increased accessibility. These genes were enriched in pathways related to FoxO signaling, autophagy, and apoptosis, whereas regions with reduced accessibility were associated with TGF-β signaling, extracellular matrix interactions, and muscle structural maintenance. RNA-seq identified 2170 differentially expressed genes (844 upregulated and 1326 downregulated), revealing a parallel transcriptional shift toward protein turnover, autophagy, and metabolic adaptation, together with repression of extracellular matrix organization, focal adhesion, integrin signaling, and cytoskeletal organization. Integrative analysis identified 113 genes with coordinated increases and 46 genes with coordinated decreases in chromatin accessibility and transcript abundance, highlighting ubiquitin-mediated proteolysis, mitophagy, FoxO signaling, and metabolic pathways as core components of the chronic cortisol response. Collectively, these findings demonstrate that sustained cortisol elevation drives coordinated epigenomic and transcriptomic remodeling of skeletal muscle, promoting a catabolic phenotype characterized by enhanced protein turnover, oxidative protein damage, and impaired structural maintenance, ultimately contributing to reduced growth and potentially compromising fish welfare under aquaculture conditions.

## 1. Introduction

Aquaculture has become a key sector for meeting the increasing global demand for aquatic dietary products, contributing substantially to food security and economic development [1]. Within this sector, salmonid aquaculture accounts for a major share of global aquaculture production, and Chile ranks among the world’s leading producers of farmed salmonids, generating substantial socioeconomic benefits at the national level [2]. Among cultured salmonids, rainbow trout (*Oncorhynchus mykiss*) is one of the most economically important and widely farmed freshwater fish species worldwide [3]. Its rapid growth, high adaptability to diverse farming conditions, and excellent nutritional and commercial value have established rainbow trout as a cornerstone of freshwater aquaculture production and a key contributor to the global salmonid industry [4]. In Chile, aquaculture production of rainbow trout reached 63,856 tonnes in 2024, accounting for approximately 5.34% of the global production of this species [5]. This figure underscores the economic importance of rainbow trout to the country and supports continued efforts to optimize its growth performance and welfare.

Intensive aquaculture systems expose fish to a wide range of stressors, including high stocking densities, handling, transportation, fluctuations in water quality, and routine husbandry procedures, all of which can activate the physiological stress response [6,7]. When this response is prolonged, chronic stress redirects energy resources away from anabolic processes toward coping mechanisms, thereby impairing productive performance, immune function, and animal welfare [6,8]. In teleosts, cortisol is the primary mediator of the stress response. This corticosteroid is released through activation of the hypothalamic–pituitary–interrenal (HPI) axis following exposure to a stressor [9]. Although an acute increase in cortisol serves an adaptive function, persistently elevated cortisol levels exert catabolic effects that can impair somatic growth. While the liver, gills, and brain have traditionally been major target tissues in fish stress physiology [10,11], skeletal muscle is particularly relevant for evaluating the long-term consequences of cortisol exposure because it constitutes the largest proportion of body mass and directly contributes to somatic growth and production performance [12]. Moreover, skeletal muscle is a major target of the catabolic actions of glucocorticoids, involving the balance between protein synthesis and degradation, autophagy, energy metabolism, and structural remodeling [13]. Unlike mammals, teleosts maintain indeterminate muscle growth throughout life through continuous hyperplasia and hypertrophy, making skeletal muscle highly responsive to environmental conditions [14]. Chronic stress diverts metabolic resources from somatic growth toward maintenance of homeostasis, resulting in reduced protein synthesis, activation of proteolytic pathways, inhibition of the growth hormone/insulin-like growth factor-1 axis, and stimulation of ubiquitin-proteasome and autophagy-lysosome systems, ultimately impairing muscle growth and fillet quality [9,15]. Understanding the molecular mechanisms underlying these effects is therefore essential for developing husbandry strategies aimed at minimizing the detrimental consequences of stress on aquaculture productivity.

Beyond its immediate physiological and genomic effects, cortisol may also contribute to epigenetic regulation by modulating mechanisms such as DNA methylation and histone modifications, potentially leading to long-lasting effects on stress adaptation [16]. In fish, exposure to stressors has been shown to alter DNA methylation and gene expression profiles, with effects that may persist over time and, in some cases, across generations [17,18,19]. These epigenetic modifications can influence chromatin organization and accessibility, thereby affecting the interaction of transcription factors with regulatory regions and ultimately contributing to the control of gene expression [20]. Genome-wide characterization of chromatin accessibility has become possible through next-generation sequencing approaches such as ATAC-seq (Assay for Transposase-Accessible Chromatin using sequencing), which enables the identification of accessible regulatory regions and provides insights into potential transcription factor binding sites and regulatory elements associated with the stress response [21,22,23,24]. While RNA-seq captures the transcriptional output associated with cortisol exposure, it does not provide direct information about the underlying chromatin regulatory landscape that may facilitate or constrain these transcriptional responses [25]. ATAC-seq complements transcriptomic analysis by identifying genomic regions with altered accessibility, including potential promoters and enhancers, thereby providing an additional regulatory layer for interpreting changes in gene expression [26]. Integrating ATAC-seq and RNA-seq can therefore distinguish transcriptional changes associated with coordinated chromatin remodeling from those that may arise through other regulatory mechanisms [27]. In mammalian skeletal muscle, integrative ATAC-seq and RNA-seq approaches have been increasingly used to identify regulatory regions, candidate genes, and transcription factors involved in muscle development, remodeling, and phenotypic regulation [28,29,30]. In contrast, the application of these integrative approaches in fish remains relatively limited.

ATAC-seq has been employed in contexts such as gill tissue and metamorphosis in flatfish [31,32], but its use in tissues undergoing stress-induced remodeling remains limited. In the liver of channel catfish (*Ictalurus punctatus*), elevated cortisol levels simultaneously reshaped the transcriptome and chromatin accessibility landscape, with differentially accessible regions predominantly located in promoters proximal to transcription start sites [33]. Similarly, recent studies in rainbow trout (*O. mykiss*) have highlighted the importance of chromatin accessibility as an epigenetic mechanism underlying cortisol-mediated stress responses [34]. ATAC-seq analyses demonstrated that acute cortisol exposure induces extensive chromatin remodeling, increasing the accessibility of regulatory regions associated with skeletal muscle adaptation. These changes promoted the enrichment of binding motifs for key stress-responsive transcription factors, including *Fos*, *AP-1*, *FoxO1/3*, *Mef2a/b/c*, *Klf5/10*, and *ATF4*, highlighting chromatin accessibility as a dynamic regulatory layer linking cortisol signaling to transcriptional responses [34]. Nevertheless, these findings were obtained under an acute cortisol exposure paradigm, and it remains unclear whether prolonged cortisol elevation induces similar or distinct patterns of chromatin accessibility and transcriptional regulation in skeletal muscle. This knowledge gap is particularly relevant because chronic stress more closely reflects the prolonged conditions that fish may experience under intensive aquaculture.

We hypothesized that sustained cortisol exposure establishes a distinct chromatin accessibility landscape associated with persistent transcriptional activation of catabolic pathways and repression of processes involved in muscle growth and structural maintenance. Elucidating these regulatory mechanisms is essential for understanding the molecular basis of stress-induced growth impairment and reduced welfare in farmed fish and may facilitate the identification of molecular markers associated with chronic stress responses. Therefore, the present study aimed to characterize the effects of prolonged cortisol exposure on chromatin accessibility and gene expression in rainbow trout skeletal muscle through the integration of ATAC-seq and RNA-seq. By simultaneously examining these two regulatory layers, this study provides new insights into the relationship between chromatin remodeling and transcriptional responses underlying skeletal muscle adaptation to sustained cortisol exposure in teleost fish.

## 2. Results

### 2.1. Transcriptomic Response of Rainbow Trout Skeletal Muscle to Chronic Cortisol Exposure

To investigate the long-term effects of cortisol on skeletal muscle, rainbow trout were subjected to a chronic cortisol administration protocol based on a single intraperitoneal injection of cortisol dissolved in coconut oil, which provides a sustained release of the hormone over time. Fish were maintained under these conditions for 14 days before tissue collection. To validate the effectiveness of the experimental treatment, plasma cortisol concentrations were measured after 14 days of cortisol administration. Cortisol-treated fish exhibited a significant increase in plasma cortisol levels compared with the vehicle-treated group (86.6 vs. 30.8 ng mL^−1^, respectively; *p*-value = 0.0339), confirming that treatment successfully induced a sustained elevation of the hormone throughout the experimental period (Figure 1A). In contrast, no significant differences in plasma LDH activity were observed between cortisol- and vehicle-treated fish (*p*-value > 0.05; Figure 1B). Chronic cortisol exposure significantly reduced growth performance over the two-week experimental period. The specific growth rate (SGR) decreased from 2.75 in the vehicle-treated group to 1.99 in cortisol-treated fish (*p*-value = 0.041) (Figure 1C). Body weight and length measurements for all experimental time points are provided in Supplementary Table S1.

To characterize the transcriptional response induced by chronic cortisol exposure, skeletal muscle RNA from rainbow trout was sequenced after 14 days of treatment. A total of six cDNA libraries (three vehicle and three cortisol-treated fish) were sequenced. A total of 398,994,962 reads were obtained. After discarding adapters and low-quality reads, we obtained 398,989,039 clean reads for RNA-seq analysis. The average mapping rate was 83.1% in the rainbow trout genome (Supplementary Table S2). Principal component analysis (PCA) clearly separated the cortisol-treated and vehicle groups, indicating that prolonged cortisol exposure induced a distinct transcriptional signature in skeletal muscle (Supplementary Figure S1A). Differential expression analysis identified 2170 differentially expressed genes (DEGs) (844 Up and 1326 Down) between vehicle and cortisol-treated fish, demonstrating extensive transcriptomic remodeling following prolonged cortisol exposure (Supplementary Figure S1B and Supplementary Table S3). Functional enrichment analysis revealed that upregulated genes were primarily associated with biological processes involved in protein turnover, including protein ubiquitination and the ubiquitin-dependent protein catabolic process, together with autophagosome assembly, transmembrane transport, and the negative regulation of apoptotic processes (Figure 2A). In contrast, downregulated genes were predominantly enriched in biological processes related to cell adhesion, extracellular matrix organization, actin filament organization, cell migration, and cell differentiation, indicating a reduction in pathways associated with tissue organization and structural maintenance (Figure 2A). Notably, regulation of transcription by RNA polymerase II and protein phosphorylation were significantly enriched in both upregulated and downregulated gene sets. KEGG pathway analysis further supported these findings. Upregulated genes were significantly enriched in pathways related to autophagy, FoxO signaling, protein processing in the endoplasmic reticulum, cell cycle, and metabolic pathways, consistent with enhanced protein turnover and metabolic adaptation (Figure 2B). Conversely, downregulated genes were mainly associated with pathways involved in ECM–receptor interaction, focal adhesion, integrin signaling, regulation of the actin cytoskeleton, and cell adhesion molecule (CAM) interactions, reflecting the suppression of extracellular matrix remodeling and cytoskeletal organization (Figure 2B). Several pathways, including metabolic pathways, MAPK signaling, calcium signaling, and cytoskeleton in muscle cells, were enriched in both transcript sets. Overall, these results suggest that chronic cortisol exposure promotes a coordinated transcriptomic shift in rainbow trout skeletal muscle, favoring protein turnover, autophagy, and metabolic adaptation, while repressing cell adhesion, extracellular matrix organization, and cytoskeletal integrity, consistent with the establishment of a chronic stress-adapted phenotype.

### 2.2. Chromatin Accessibility Remodeling Induced by Chronic Cortisol Exposure in Rainbow Trout Skeletal Muscle

ATAC-seq libraries were generated from skeletal muscle of vehicle- and cortisol-treated rainbow trout using three biological replicates per condition. Sequencing yielded a total of 500,600,152 paired-end reads, of which 500,181,676 were retained after adapter trimming and quality filtering. The processed reads had an average length of 100 bp and showed a mean mapping rate of approximately 94% against the *O. mykiss* reference genome, indicating high-quality libraries suitable for downstream chromatin accessibility analyses (Supplementary Table S4). Library size distribution analysis showed the characteristic enrichment of short nucleosome-free fragments together with periodic nucleosome-associated fragments, reflecting appropriate chromatin fragmentation and nucleosome organization across all biological replicates (Supplementary Figure S2A). The heatmap showed that ATAC-seq accessibility for cortisol groups was slightly higher than for vehicle groups, suggesting a dynamic regulation of chromatin accessibility induced by cortisol (Supplementary Figure S2B). Genome-wide annotation of accessible chromatin regions revealed that peaks were predominantly distributed within intergenic regions and introns (Figure 3A). Analysis of chromatin accessibility across chromosomes showed that accessible regions were distributed throughout the rainbow trout genome under both experimental conditions. However, the cortisol-treated group exhibited a greater number of accessible peaks across most chromosomes (Figure 3B). Consistent with this observation, overlap analysis identified 317,530 shared peaks, 95,711 vehicle-specific peaks, and 126,780 cortisol-specific peaks, demonstrating that chronic cortisol exposure generated a substantial number of treatment-specific accessible chromatin regions (Figure 3C). Furthermore, annotation of treatment-specific peaks identified 1580 differentially accessed genes (DAGs) in the vehicle group and 3393 DAGs in the cortisol group. Transcription factor binding motif enrichment analysis was performed by comparing differentially accessible chromatin regions identified in rainbow trout skeletal muscle against vertebrate transcription factor motif databases. A total of 67 significantly enriched transcription factor motifs were identified (*p*-value < 0.01). The most highly enriched motifs corresponded to members of the *CREB/ATF* family, *C/EBP* family, *NF-κB*, *SRF*, *TEAD*, *STAT*, *FoxO1*, *FoxO3*, and *ATF4* (Figure 3D).

To investigate the biological significance of cortisol-induced chromatin remodeling, functional enrichment analyses were performed using DAGs. GO Biological Process analysis revealed that regions exhibiting increased accessibility following cortisol treatment were exclusively enriched in biological processes related to intracellular signal transduction, positive regulation of transcription by RNA polymerase II, intracellular protein transport, mRNA processing, RNA splicing, ubiquitin-dependent protein catabolic processes, protein dephosphorylation, and transmembrane transport (Figure 4A). Conversely, regions showing higher accessibility in the vehicle group were exclusively associated with signal transduction, cell differentiation, G protein-coupled receptor signaling pathway, apoptotic process, actin filament organization, brain development, and positive regulation of DNA-templated transcription (Figure 4A). Regulation of transcription by RNA polymerase II, protein phosphorylation, negative regulation of transcription by RNA polymerase II, and multicellular organism development were significantly enriched in both groups (Figure 4A). KEGG pathway analysis further supported these findings. Regions with increased accessibility following cortisol treatment were exclusively enriched in pathways related to protein processing in the endoplasmic reticulum, insulin signaling, FoxO signaling, autophagy, and apoptosis, consistent with enhanced stress adaptation, protein turnover, and metabolic remodeling (Figure 4B). In contrast, regions that remained more accessible in the vehicle group were exclusively enriched in Notch signaling, PPAR signaling, ECM–receptor interaction, TGF-β signaling, and cardiac muscle contraction (Figure 4B). Several pathways were enriched in both conditions, including metabolic pathways, MAPK signaling, cytoskeleton in muscle cells, focal adhesion, calcium signaling, and adrenergic signaling in cardiomyocytes (Figure 4B). Overall, these findings suggest that prolonged cortisol exposure extensively reshapes the chromatin landscape of rainbow trout skeletal muscle by increasing the accessibility of regulatory regions involved in protein turnover, intracellular signaling, transcriptional regulation, and metabolic adaptation, while reducing the accessibility of chromatin associated with cell communication, extracellular matrix interactions, developmental signaling, and muscle structural homeostasis.

### 2.3. Integrative Analysis of ATAC-Seq/RNA-Seq and Validation

Integrative findings of ATAC-seq and RNA-seq showed an overlap of 113 genes between up-regulated DEGs and up-regulated DAGs, and 46 genes between down-regulated DEGs and down-regulated DAGs (Supplementary Table S5 and Supplementary Figure S3). To investigate the biological significance of cortisol-induced chromatin remodeling, Gene Ontology (GO) Biological Process enrichment analysis was performed. Genes exhibiting increased accessibility and expression following cortisol treatment were exclusively enriched in biological processes associated with glyceraldehyde-3-phosphate biosynthesis, glycerol catabolism, carboxylic acid metabolism, glycogen metabolism, lipid homeostasis, protein demethylation, amino acid transmembrane transport, and endoplasmic reticulum-to-Golgi vesicle-mediated transport (Figure 5A). In contrast, regions showing higher accessibility and expression in the vehicle group were exclusively enriched in biological processes related to actin filament organization, cell motility, phagocytosis, transmembrane transport, bicarbonate transport, regulation of intracellular pH, and positive regulation of cell differentiation (Figure 5A). KEGG pathway enrichment analysis revealed distinct functional signatures between chromatin regions exhibiting increased accessibility following cortisol treatment and those showing higher accessibility in the vehicle group. Genes with increased accessibility and expression in the cortisol group were exclusively enriched in pathways associated with metabolic pathways, mitophagy, ubiquitin-mediated proteolysis, insulin signaling, apoptosis, and FoxO signaling, indicating enhanced metabolic remodeling, protein turnover, and stress adaptation (Figure 5B). Additional pathways specifically enriched in the cortisol group included focal adhesion, reflecting changes in intracellular signaling and cellular remodeling. In contrast, genes that remained more accessible and expressed in the vehicle group were exclusively enriched in pathways related to efferocytosis, cardiac muscle contraction, bacterial invasion of epithelial cells, GnRH signaling, cornified envelope formation, cell adhesion molecule (CAM) interaction, and tight junction, suggesting that chronic cortisol exposure reduces chromatin accessibility at genes involved in cell communication, tissue organization, and structural homeostasis (Figure 5B). Several pathways were enriched in both groups, including metabolic pathways, adrenergic signaling in cardiomyocytes, calcium signaling, Wnt signaling, and cadherin signaling (Figure 5B).

For RT-qPCR validation, genes were selected based on two main criteria: (i) concordant changes in chromatin accessibility and transcript abundance between ATAC-seq and RNA-seq analyses, and (ii) their functional relevance to biological processes associated with cortisol-induced skeletal muscle remodeling. Based on these criteria, four genes were selected: *foxo3b* (ENSOMYG00000062959), associated with muscle catabolism; *cullin-3* (ENSOMYG00000045182), involved in ubiquitin-mediated proteolysis; *samtor* (ENSOMYG00000014559), related to nutrient sensing; and *bcl2* (ENSOMYG00000043056), involved in the regulation of apoptosis. RT-qPCR analysis confirmed the expression patterns identified by RNA-seq, with *foxo3b* and *cullin-3* being significantly upregulated and *samtor* significantly downregulated in cortisol-treated fish, demonstrating a strong positive correlation between both approaches (r = 0.9246; Figure 6A). No significant changes in *bcl2* expression were detected between cortisol-treated and control fish. To determine whether sustained cortisol exposure was also associated with cellular oxidative damage, DNA and protein oxidation were evaluated in skeletal muscle. Although AP site abundance tended to increase in cortisol-treated fish, no significant differences were detected compared with the vehicle group (Figure 6B; *p*-value > 0.05). In contrast, protein carbonyl content was significantly increased following chronic cortisol exposure (Figure 6C; *p*-value = 0.038), indicating enhanced oxidative modification of skeletal muscle proteins. Together, these results show that prolonged cortisol exposure is associated with protein oxidative damage.

## 3. Discussion

The present study provides the first integrative analysis of chromatin accessibility and transcriptomic remodeling induced by prolonged cortisol exposure in the skeletal muscle of rainbow trout. By combining ATAC-seq and RNA-seq following fourteen days of sustained cortisol elevation, we demonstrate that chronic glucocorticoid stimulation induces extensive epigenomic reprogramming accompanied by coordinated transcriptional changes that promote a persistent catabolic phenotype. Unlike the transient molecular responses previously observed following acute cortisol exposure [34], prolonged treatment produced widespread chromatin remodeling associated with activation of ubiquitin-mediated proteolysis, autophagy, mitophagy, apoptosis, and FoxO signaling, while simultaneously repressing extracellular matrix organization, focal adhesion, integrin signaling, cytoskeletal organization, and TGF-β signaling. These findings indicate that chronic cortisol exposure represents a distinct biological state in which stable epigenetic remodeling reinforces long-term metabolic adaptation at the expense of muscle growth and structural maintenance.

Cortisol is the principal glucocorticoid regulating the teleost stress response through activation of glucocorticoid receptors, coordinating metabolic adjustments that restore homeostasis during acute stress [35,36]. However, persistent activation of the HPI axis imposes substantial energetic costs that impair growth, immune competence, and tissue integrity [37]. One of the most widely adopted approaches for investigating the physiological consequences of chronic stress in teleost fish has been the administration of slow-release intraperitoneal cortisol implants [38]. Unlike acute stress paradigms, which are often confounded by repeated handling and activation of multiple neuroendocrine pathways, slow-release implantation systems provide a controlled and sustained delivery of lipophilic compounds, including cortisol, allowing researchers to investigate their long-term physiological effects under well-controlled experimental conditions, minimizing handling-induced stress [39]. One of the earliest studies investigating the chronic physiological effects of cortisol in teleosts was conducted by Pickering and Duston (1983) in brown trout (*Salmo trutta*), who established a slow-release intraperitoneal implantation method using cocoa butter capable of maintaining sustained physiological plasma cortisol concentrations for several weeks [40]. They demonstrated that prolonged cortisol exposure reduced fish condition and growth while increasing susceptibility to fungal (Saprolegnia) and bacterial (*Aeromonas salmonicida*) infections, providing the first evidence that chronic cortisol elevation alone can compromise fish health and growth, similar to our observations [40]. Building on these initial observations in brown trout, subsequent studies extended the use of slow-release cortisol implants to investigate the long-term physiological consequences of sustained glucocorticoid exposure in other teleost species. In rainbow trout (*O. mykiss*), Gregory and Wood (1999) demonstrated that sustained cortisol elevation reduced voluntary feed intake, impaired growth performance, and increased the energetic cost of growth independently of social interactions, introducing the concept that chronic cortisol redirects energy allocation away from somatic growth toward maintenance processes [41]. Similar effects were later reported in rainbow trout by Madison et al. (2015) [42], who showed that prolonged cortisol exposure suppresses growth through sustained induction of *igfbp1*, disruption of the GH/IGF axis, and mobilization of carbohydrate and lipid reserves. More recently, Pfalzgraff et al. (2022) and Vargas-Chacoff et al. (2021), working in rainbow trout (*O. mykiss*) and Atlantic salmon (*Salmo salar*), respectively, demonstrated that chronic cortisol suppresses growth even when food intake is maintained, decreases digestive efficiency, and increases the energetic cost of tissue accretion, promoting a systemic catabolic state, similar to our results [43,44]. As the chronic cortisol model became increasingly established, research shifted from whole-animal physiology toward elucidating the molecular mechanisms underlying sustained glucocorticoid signaling. Teles et al. (2013) demonstrated that prolonged cortisol exposure differentially regulates glucocorticoid and mineralocorticoid receptor expression among tissues, indicating that tissue sensitivity to cortisol is dynamically modulated during chronic exposure [45]. Similarly, Cortés et al. (2013) subsequently showed that cortisol alone is sufficient to induce coordinated transcriptional changes in innate immune pathways independently of other neuroendocrine components of the stress response [46]. Building upon these findings, Aedo et al. (2021) demonstrated that after 72 h of cortisol treatment in gilthead seabream (*Sparus aurata*), genomic, but not membrane-initiated, glucocorticoid signaling promotes hepatic gluconeogenesis through the induction of *pepck* and *g6pc*, while simultaneously increasing the expression of the muscle atrogenes *atrogin-1* and *murf1*, thereby establishing genomic GR signaling as the predominant mechanism underlying the metabolic and catabolic responses to prolonged cortisol exposure [47]. These observations are highly consistent with our results, which revealed enhanced chromatin accessibility and transcriptional activation of pathways associated with gluconeogenesis, FoxO signaling, autophagy, and ubiquitin-mediated proteolysis in skeletal muscle, suggesting that sustained cortisol exposure orchestrates a coordinated genomic program promoting energy mobilization and muscle protein degradation. The application of transcriptomic and multi-omics approaches has subsequently expanded this mechanistic framework. Teles et al. (2013) reported extensive hepatic transcriptional remodeling involving carbohydrate metabolism, proteolysis, apoptosis, and immune suppression in *S. aurata* [48]. More recently, Blanco et al. (2024) demonstrated profound hypothalamic metabolic remodeling associated with an “energy excess” signature and appetite suppression [49], whereas Schmidt et al. (2026) and Virtanen et al. (2023) showed that chronic cortisol exposure or prolonged stress induces coordinated transcriptional reprogramming in immune tissues, impairs wound healing, promotes inflammation, and compromises fish welfare [50,51]. Together with our integrated ATAC-seq and RNA-seq analyses, these studies indicate that chronic cortisol signaling extends beyond isolated metabolic pathways to drive extensive tissue-specific transcriptional and epigenomic remodeling across multiple physiological systems. Previous transcriptomic studies demonstrated that cortisol rapidly activates catabolic pathways while suppressing anabolic processes in fish skeletal muscle [52,53,54], yet the epigenetic mechanisms responsible for maintaining these responses during chronic stress remained largely unknown.

Our results indicate that prolonged cortisol exposure induces extensive genome-wide chromatin remodeling, characterized by numerous cortisol-specific accessible regions and differentially accessible genes, thereby establishing a regulatory landscape that may sustain stress-responsive transcriptional programs during chronic stress. Similar large-scale chromatin remodeling has been described in mammalian systems, where glucocorticoid receptor binding promotes nucleosome displacement, enhancer activation, and recruitment of chromatin-remodeling complexes [55,56,57]. Interestingly, studies in mammals have demonstrated that chromatin accessibility undergoes extensive remodeling during skeletal muscle atrophy. Using single-myofiber transcriptomic and epigenomic sequencing, Sahinyan et al. reported that differentially accessible regions were predominantly located within intronic and distal intergenic regions corresponding to enhancer elements, similar to our observations [58,59]. Furthermore, these accessible regions largely overlapped with H3K27ac-enriched sites, indicating that enhancer activation is closely associated with chromatin remodeling during muscle adaptation. Although comparable studies in teleosts are scarce, our results suggest that these conserved mechanisms also regulate long-term stress adaptation in fish skeletal muscle. Importantly, comparison with our previous study of acute cortisol exposure reveals a clear temporal progression in glucocorticoid action [34]. Three hours after cortisol administration, chromatin remodeling was primarily associated with immediate stress-responsive transcription factors, including *AP-1*, *ATF4*, *KLF* family members, *MEF2* proteins, and *FoxO*, together with early activation of autophagy-related genes [34]. In contrast, prolonged cortisol exposure produced a much broader remodeling of chromatin accessibility associated with sustained activation of catabolic pathways and repression of structural maintenance. These observations indicate that the acute response evolves into a stable epigenetic program capable of maintaining altered gene expression during prolonged endocrine stimulation [60]. The coordinated enrichment of FoxO signaling, autophagy, ubiquitin-mediated proteolysis, apoptosis, and protein processing strongly supports the establishment of a persistent catabolic state. FoxO transcription factors are master regulators of skeletal muscle atrophy, integrating glucocorticoid signaling with insulin signaling, oxidative stress, and nutrient availability [61]. Activation of FoxO stimulates expression of numerous atrogenes involved in protein degradation and autophagy, including *fbxo32*, *trim63*, *bnip3*, and *lc3* [62]. Our data indicate that prolonged chromatin opening surrounding these regulatory pathways reinforces sustained activation of protein degradation, suggesting that epigenetic remodeling contributes directly to maintaining muscle catabolism during chronic stress. Integration of ATAC-seq and RNA-seq provides additional insight into the mechanisms underlying this response. Although only 113 genes showed coordinated increases in chromatin accessibility and expression, whereas 46 genes exhibited coordinated decreases, this relatively modest overlap is consistent with the complexity of gene regulation. Changes in chromatin accessibility can establish a permissive regulatory state but are not necessarily sufficient to induce simultaneous changes in transcript abundance, as transcription also depends on transcription factor occupancy, enhancer–promoter interactions, chromatin architecture, histone modifications, and DNA methylation [63]. Conversely, changes in gene expression may occur without detectable alterations in local chromatin accessibility and may also be influenced by post-transcriptional mechanisms, including mRNA stability and degradation [64]. Indeed, integrative ATAC-seq and RNA-seq analyses in MCF-7 breast carcinoma cells exposed to retinoic acid or TGF-β showed that substantial changes in gene expression can occur with little or no detectable change in local chromatin accessibility, supporting the notion that accessibility and transcriptional responses are not necessarily tightly coupled under specific perturbations [65]. Temporal dynamics may further contribute to the limited overlap observed at 14 days, as some accessibility changes could represent regulatory priming whose transcriptional consequences emerge at later stages, whereas others may reflect persistent chromatin states established by earlier transcriptional responses. Thus, the concordant subset of DAGs and DEGs likely represents genes for which sustained cortisol exposure is associated with a particularly strong coupling between chromatin remodeling and transcriptional regulation [63]. Among these overlapping genes, enrichment of ubiquitin-mediated proteolysis, autophagy, mitophagy, and metabolic pathways highlights protein turnover as a principal target of chronic glucocorticoid regulation [66]. The ubiquitin-proteasome system and the autophagy-lysosome pathway normally cooperate to maintain protein quality control, but persistent glucocorticoid exposure shifts this balance toward extensive protein degradation [67]. Similarly, activation of mitophagy suggests that chronic cortisol promotes mitochondrial remodeling in addition to protein degradation [68]. Although these responses initially preserve cellular homeostasis by removing damaged proteins and organelles, prolonged activation ultimately reduces muscle mass and growth potential. Conversely, repression of extracellular matrix organization, focal adhesion, integrin signaling, regulation of the actin cytoskeleton, and TGF-β signaling suggests that chronic cortisol suppresses the structural mechanisms required for muscle maintenance [66]. Reduced accessibility and expression of genes involved in these pathways indicate that chronic glucocorticoid exposure compromises both intracellular metabolism and tissue architecture [69]. Such coordinated suppression likely contributes to the reduced growth rate observed throughout the experimental period and may explain the impaired regenerative capacity frequently reported in chronically stressed fish. Interestingly, several metabolic pathways were enriched in both upregulated and downregulated datasets, indicating that chronic cortisol does not simply increase or decrease metabolism but rather redistributes metabolic priorities. Energy is preferentially allocated toward protein turnover, amino acid mobilization, and cellular maintenance while energetically expensive processes such as extracellular matrix synthesis and tissue growth are suppressed [70]. This metabolic reallocation is consistent with the concept of allostasis, whereby organisms prioritize survival over growth under prolonged stress [71].

To validate the integrated ATAC-seq and RNA-seq analyses, we selected four genes showing concordant changes in chromatin accessibility and gene expression that represent key pathways involved in cortisol-induced skeletal muscle remodeling: nutrient sensing (*samtor*), ubiquitin-mediated proteolysis (*cullin-3*), muscle catabolism (*foxo3b*), and apoptosis (*bcl2*). Chronic cortisol exposure significantly upregulated *foxo3b* and *cullin-3* and downregulated *samtor*, whereas *bcl2* showed no significant changes in expression by RT-qPCR. The increased expression of *foxo3b* is consistent with the enrichment of the FoxO signaling pathway and supports the activation of transcriptional programs controlling ubiquitin-mediated proteolysis and autophagy, two hallmark processes of skeletal muscle atrophy in fish [72]. Likewise, the upregulation of *cullin-3* suggests an enhanced protein degradation capacity in cortisol-treated muscle and is consistent with previous reports describing *cullin-3* induction in zebrafish skeletal muscle during acute cold stress, supporting its broader role in stress-induced muscle remodeling [73]. In contrast, the reduced expression of *samtor* indicates altered nutrient sensing and metabolic signaling that may contribute to the shift from anabolic to catabolic metabolism during chronic stress. Interestingly, these findings contrast with our previous study, in which acute cortisol exposure induced the upregulation of *samtor* in rainbow trout skeletal muscle, as part of an early adaptive response associated with mTORC1 inhibition and autophagy activation [34]. Similarly, although the downregulation of the anti-apoptotic gene *bcl2* did not reach statistical significance by RT-qPCR, a decreasing trend consistent with the RNA-seq results was observed, suggesting a potential reduction in the capacity to maintain cellular homeostasis, potentially facilitating the coordinated regulation of apoptosis and autophagy, a response previously associated with crowding stress-induced muscle atrophy in fine flounder and rainbow trout [13,74]. Together, these results support the activation of an integrated catabolic program underlying chronic cortisol-induced skeletal muscle remodeling. The assessment of oxidative damage provided an additional physiological validation of the molecular signature associated with cortisol-induced muscle atrophy. Chronic cortisol exposure significantly increased protein carbonylation in skeletal muscle, whereas DNA oxidative damage, assessed through AP-site abundance, remained unchanged. This preferential accumulation of oxidatively modified proteins is consistent with the transcriptomic and chromatin accessibility profiles observed in our study, which revealed coordinated enrichment of pathways related to ubiquitin-mediated proteolysis, autophagy, and FoxO signaling. Oxidative modification can alter protein structure and function, promoting the recognition and removal of damaged proteins by cellular protein quality-control systems and thereby contributing to increased protein turnover [75]. Importantly, comparable oxidative responses have been reported in fish subjected to chronic stressors. Prolonged crowding in rainbow trout induces oxidative damage in skeletal muscle together with molecular signatures of muscle atrophy [76], while environmental stressors such as temperature can similarly disrupt redox homeostasis and promote oxidative damage [77]. Several limitations of the present study should be acknowledged. First, chromatin accessibility and transcriptional responses were evaluated only after 14 days of cortisol exposure, limiting our ability to resolve the temporal dynamics of these regulatory changes. Indeed, time-course integration of ATAC-seq and RNA-seq in other biological models has revealed sequential changes in gene expression accompanied by dynamic patterns of chromatin accessibility, highlighting the importance of temporal resolution for interpreting their regulatory relationship [78]. Second, because the fish were at an early juvenile stage and lacked clearly distinguishable external sexual characteristics, sex was not determined. This should be considered when interpreting our results, as integrative ATAC-seq and RNA-seq analyses in fish gonads have identified sex-associated differences in gene expression together with corresponding changes in open chromatin regions [79]. Thus, potential sex-dependent differences in the molecular response to prolonged cortisol exposure cannot be excluded.

## 4. Materials and Methods

### 4.1. Experimental Protocol

All experimental procedures were approved by the Bioethics Committee of Universidad Andrés Bello and the Chilean National Commission for Scientific and Technological Research (protocol code 012/2023) and were conducted in accordance with institutional guidelines for the care and use of experimental animals. Juvenile rainbow trout (*Oncorhynchus mykiss*; 18.8 ± 5.5 g body weight; 12.6 ± 1.3 cm total length) were obtained from Pisciculture Río Blanco (Los Andes, Valparaíso Region, Chile). Sex was not determined because the fish were at an early juvenile developmental stage and did not exhibit clearly distinguishable external sexual characteristics. Fish were acclimated under controlled laboratory conditions at 14 ± 1 °C, under a 12 h light:12 h dark photoperiod, with continuous aeration and supplied with commercial pellets once daily. Fish were maintained in 40 L tanks, with three fish per tank, under a continuous flow-through water system. Feeding was suspended 24 h before the experimental treatment. Fish were randomly assigned to either the vehicle or cortisol treatment (three individual fish per group (n = 3 biological replicates)). Prior to treatment (day 0), individual body weight and total length were recorded. Fish were then anesthetized with benzocaine (25 mg L^−1^) and received a single intraperitoneal injection of either coconut oil (vehicle) or cortisol (Sigma-Aldrich, St. Louis, MO, USA; catalog number SMB00980) dissolved in coconut oil (10 mg mL^−1^) at a final dose of 50 μg cortisol g^−1^ body weight, following the protocol previously described by Aedo et al. (2021) [47]. This formulation provides a sustained release of cortisol and has been widely used to experimentally mimic chronic glucocorticoid exposure in teleost fish. To monitor the effects of prolonged cortisol exposure on somatic growth, body weight and total length were measured again on days 7 and 14 of the experimental period. At the end of the 14-day treatment, fish were euthanized with an overdose of benzocaine (300 mg L^−1^). Blood from the caudal vessel was collected using a 1 mL heparinized (10 mg/mL) syringe, then centrifuged at 2000× *g* for 5 min at 4 °C to collect plasma, which was immediately stored at −80 °C. Myotome skeletal muscle was sampled from all fish, specifically a cross-section from the epaxial area. Finally, samples were frozen by liquid nitrogen and stored at −80 °C until processed.

### 4.2. Endocrinological and Oxidative Stress Parameters

Plasma cortisol concentrations and lactate dehydrogenase (LDH) activity were quantified using a Cayman Chemical kit (Ann Arbor, MI, USA; catalog Number 500370) and a Valtek Diagnostics Kit (Santiago, Chile; catalog Number 210-300), respectively, according to the manufacturers’ instructions. To evaluate oxidative damage at the DNA level, the abundance of apurinic/apyrimidinic (AP) sites was determined in genomic DNA isolated from skeletal muscle. Briefly, DNA was extracted from 20 mg of tissue using DNAzol reagent (Invitrogen, Carlsbad, CA, USA; catalog number 10503027) according to the supplier’s protocol. DNA yield and purity were assessed using an Epoch Spectrophotometer System (BioTek, Winooski, VT, USA). The resulting DNA samples were analyzed for AP-site content with the OxiSelect Oxidative DNA Damage Quantification Kit (Cell Biolabs, San Diego, CA, USA; catalog number STA-320), following the procedure recommended by the manufacturer. Oxidative modification of proteins was assessed by measuring protein carbonyl levels. Total proteins were obtained from 50 mg of skeletal muscle homogenized in 1 mL of lysis buffer containing 50 mM Tris-HCl (pH 7.4), 150 mM NaCl, 1 mM EDTA, and 1% NP-40. Following centrifugation at 12,000× *g*, the soluble protein fraction was recovered and maintained at 4 °C. Protein concentration was determined using the Pierce BCA Protein Assay Kit (Thermo Scientific, Bannockburn, IL, USA; catalog number 23225). Carbonyl content was subsequently measured with the OxiSelect Protein Carbonyl Spectrophotometric Assay (Cell Biolabs, CA, USA; catalog number STA-315), in accordance with the manufacturer’s protocol.

### 4.3. RNA Isolation, Library Construction and Sequencing

Total RNA was extracted from approximately 100 mg of epaxial skeletal muscle using TRIzol Reagent (Invitrogen, Carlsbad, CA, USA; catalog number 15596026) according to the manufacturer’s instructions. RNA purity was determined using a NanoDrop spectrophotometer (Epoch, BioTek, Winooski, VT, USA), whereas RNA integrity was assessed using a Fragment Analyzer (Advanced Analytical Technologies, Ames, IA, USA). Only high-quality RNA samples, with an RNA integrity number (RIN) ≥ 7.0 and an A260/A280 ratio between 1.8 and 2.1, were used for subsequent analyses. RNA-seq libraries were prepared from 1 μg of total RNA using the TruSeq RNA Sample Preparation Kit v2 (Illumina, San Diego, CA, USA; catalog number RS-122-2001) following the manufacturer’s recommendations. Briefly, poly(A)+ RNA was isolated, fragmented, reverse-transcribed into first-strand cDNA, followed by second-strand synthesis, end repair, 3′ adenylation, adapter ligation, and PCR amplification according to the standard TruSeq protocol. Library concentration was determined by fluorometry using the Qubit dsDNA High Sensitivity Assay (Invitrogen), and library integrity and fragment size distribution were evaluated with the Fragment Analyzer prior to sequencing. The six indexed libraries (three biological replicates per experimental group) were sequenced by Macrogen Inc. (Seoul, Republic of Korea) using the Illumina NovaSeq X platform (Illumina, Inc., San Diego, CA, USA) with a paired-end 150 bp (2 × 150 bp) sequencing strategy.

### 4.4. RNA-Seq Data Processing and Differential Expression Analysis

RNA-seq reads were processed and analyzed with CLC Genomics Workbench v23 (CLC bio, Qiagen, TX, USA). Sequencing quality was initially evaluated using the quality assessment tools implemented in the software. Adapter contamination and low-quality nucleotides were subsequently removed with the Trim Sequences module, applying a Phred score cutoff of 20; reads shorter than 50 nucleotides after trimming were excluded from further analyses. The resulting high-quality reads were aligned against the *Oncorhynchus mykiss* reference genome USDA_OmykA_1.1 (RefSeq accession GCF_013265735.2) using the RNA-Seq Analysis module. Alignment settings included a mismatch cost of 2, insertion and deletion costs of 3, a length fraction of 0.8, and a similarity fraction of 0.8, and only uniquely aligned reads were considered for subsequent analyses. Gene and transcript features were defined according to the GTF annotation file corresponding to the reference genome. Transcript abundance was estimated using the Expression Value Calculation tool and reported as Transcripts Per Million (TPM) and Reads Per Kilobase of transcript per Million mapped reads (RPKM). Differences in sequencing depth and gene-specific effects among libraries were accounted for using the median-of-ratios normalization approach. Differential expression was assessed with the Empirical Analysis of DGE module using a negative binomial generalized linear model. Principal component analysis (PCA) was additionally performed to examine sample clustering and identify potential batch-related variation. Genes were classified as differentially expressed when they exhibited a false discovery rate (FDR) < 0.05 and an absolute fold change ≥ 2. Functional annotation and enrichment analyses were performed using the Database for Annotation, Visualization and Integrated Discovery (DAVID v6.8) [80]. Gene Ontology (GO) enrichment analyses were performed separately for Biological Process (BP), Molecular Function (MF), and Cellular Component (CC) categories using the default DAVID parameters (minimum gene count = 2; EASE score = 0.1), considering terms with *p*-value < 0.01 as significantly enriched. Kyoto Encyclopedia of Genes and Genomes (KEGG) pathway enrichment analysis was subsequently performed to identify biological pathways significantly affected by chronic cortisol exposure.

### 4.5. Nuclear Isolation and ATAC-Seq Library Construction

Nuclei were isolated from approximately 300 mg of epaxial skeletal muscle collected from three biological replicates per experimental condition. Frozen tissues were mechanically minced in ice-cold lysis buffer (10 mM Tris-HCl, 10 mM NaCl, 3 mM MgCl_2_ and 0.1% NP-40) using sterile scissors, followed by gentle homogenization with disposable pestles. The homogenate was diluted in washing buffer (PBS supplemented with 2% bovine serum albumin) and sequentially filtered through 70-μm and 40-μm cell strainers to remove tissue debris. Samples were centrifuged at 4 °C, and the nuclear pellet was washed with PBS containing RNase inhibitor before a second centrifugation step. Nuclear integrity and viability were evaluated by trypan blue staining, and the final suspension was adjusted to 60,000 nuclei mL^−1^ using a Neubauer counting chamber. ATAC-seq libraries were generated using the Zymo-Seq ATAC Library Kit (Zymo Research, Irvine, CA, USA; catalog number D5458) according to the manufacturer’s instructions. Briefly, isolated nuclei were subjected to Tn5 transposase-mediated tagmentation, simultaneously fragmenting accessible chromatin regions and incorporating sequencing adapters. Libraries were subsequently amplified by PCR under the following cycling conditions: 72 °C for 5 min; 98 °C for 30 s; followed by 15 cycles of 98 °C for 10 s, 63 °C for 10 s, and 72 °C for 30 s. Library concentration was determined using the Qubit dsDNA High Sensitivity Assay (Invitrogen, Carlsbad, CA, USA), whereas library integrity and fragment size distribution were evaluated by capillary electrophoresis using a Fragment Analyzer Automated CE System (Advanced Analytical Technologies, Ames, IA, USA). Indexed libraries were sequenced by Macrogen Inc. (Seoul, Republic of Korea) on an Illumina NovaSeq 6000 platform using a paired-end 150 bp (2 × 150 bp) sequencing strategy.

### 4.6. ATAC-Seq Data Processing and Downstream Analyses

Raw ATAC-seq data were processed using the nf-core/atacseq pipeline (v2.1.2). Briefly, sequencing quality was initially assessed using FastQC (v0.11.9), and adapter sequences together with low-quality bases were removed using Trim Galore! (v0.6.10) with default parameters. Post-trimming quality was subsequently evaluated using FastQC to ensure high-quality reads for downstream analyses. Filtered reads were aligned against the rainbow trout reference genome (*Oncorhynchus mykiss*, USDA_OmykA_1.1; RefSeq accession GCF_013265735.2) using Bowtie2 (v2.5.1) [81]. Accessible chromatin regions (peaks) were identified using MACS3 (v3.0.0) [82], and consensus peak sets were generated across biological replicates. Peak annotation relative to genomic features, including promoters/transcription start sites (TSS), exons, introns, transcription termination sites (TTS), and intergenic regions, was performed using HOMER (v4.11) [83]. Genomic interval manipulations and peak overlap analyses were carried out using BEDTools (v2.31.0). Read counts associated with consensus peaks were quantified using featureCounts, and normalized read coverage tracks (BigWig files) were generated automatically by the pipeline for genome-wide visualization [84]. Differential chromatin accessibility analyses were performed using the DESeq2 package in R 4.6.1, considering differentially accessible regions with an adjusted *p*-value (Benjamini–Hochberg FDR) < 0.05 as statistically significant [85]. Genes associated with differentially accessible regions were subjected to functional enrichment analysis using the Database for Annotation, Visualization and Integrated Discovery (DAVID v6.8) [80]. Gene Ontology (GO) enrichment analyses were performed using the default DAVID parameters (minimum gene count = 2; EASE score = 0.1), considering terms with *p*-value < 0.01 as significantly enriched. Kyoto Encyclopedia of Genes and Genomes (KEGG) pathway enrichment analysis was subsequently performed to identify biological pathways associated with chronic cortisol-induced chromatin remodeling.

### 4.7. Quantitative Real-Time PCR Validation

Total RNA was isolated from skeletal muscle using TRIzol reagent (Ambion, Carlsbad, CA, USA; catalog number 15596026) according to the manufacturer’s recommendations. RNA concentration was measured with a NanoDrop spectrophotometer, while RNA integrity was evaluated by electrophoresis on a 1.2% formaldehyde–agarose gel. For first-strand cDNA synthesis, 1 μg of total RNA was reverse transcribed with the ImProm-II Reverse Transcription System (Promega, Madison, WI, USA; catalog number A3800) following the supplier’s protocol. Primers for the selected target genes were designed and evaluated using OligoAnalyzer 3.1. Quantitative real-time PCR (RT-qPCR) was carried out using a Stratagene MX3000P system (Stratagene, La Jolla, CA, USA). Each 20 μL reaction contained 7.5 μL of 2× Brilliant^®^ II SYBR^®^ Green Master Mix (Agilent Technologies, Santa Clara, CA, USA; catalog number 600828), 6 μL of 40-fold diluted cDNA, and each primer at a final concentration of 250 nM. No-template (NTC) and no-reverse-transcriptase (noRT) controls were included to detect potential reagent contamination and genomic DNA amplification, respectively. Primer sequences are provided in Supplementary Table S6. Reactions were performed in triplicate using an initial denaturation step at 95 °C for 2 min, followed by 40 cycles consisting of denaturation at 95 °C for 30 s, primer-specific annealing at 54–60 °C for 30 s, and extension at 72 °C for 30 s. Amplification specificity was verified by melting-curve analysis following each run. Relative transcript abundance was calculated using the ΔΔ^Ct^ method [86], with *β-actin* (*actβ*) and 40S ribosomal protein S30 (*fau*) as reference genes. Expression values were normalized to the vehicle-treated group and reported as fold change.

### 4.8. Statistical Analysis

All data, including gene expression, physiological parameters, growth performance, and oxidative damage measurements, are presented as mean ± standard error of the mean (SEM). Statistical differences between the vehicle- and cortisol-treated groups were evaluated using an unpaired two-tailed Student’s *t*-test. Statistical analyses were performed using GraphPad Prism 8.0 (GraphPad Software, Inc., San Diego, CA, USA). Differences were considered statistically significant at *p*-value < 0.05.

## 5. Conclusions

Prolonged cortisol exposure induces coordinated epigenomic and transcriptomic remodeling that promotes a stable catabolic phenotype in rainbow trout skeletal muscle. Sustained activation of protein degradation, autophagy, mitophagy, and metabolic adaptation is accompanied by repression of extracellular matrix organization, cytoskeletal integrity, and tissue remodeling, revealing that chronic stress extends beyond transient transcriptional regulation to establish long-term epigenetic programs. These findings identify chromatin remodeling as a fundamental mechanism underlying skeletal muscle adaptation to chronic stress and provide new insights into the molecular basis of growth impairment in teleosts, while offering promising targets for the development of biomarkers aimed at improving fish welfare and resilience in aquaculture.

## Figures and Tables

**Figure 1:**
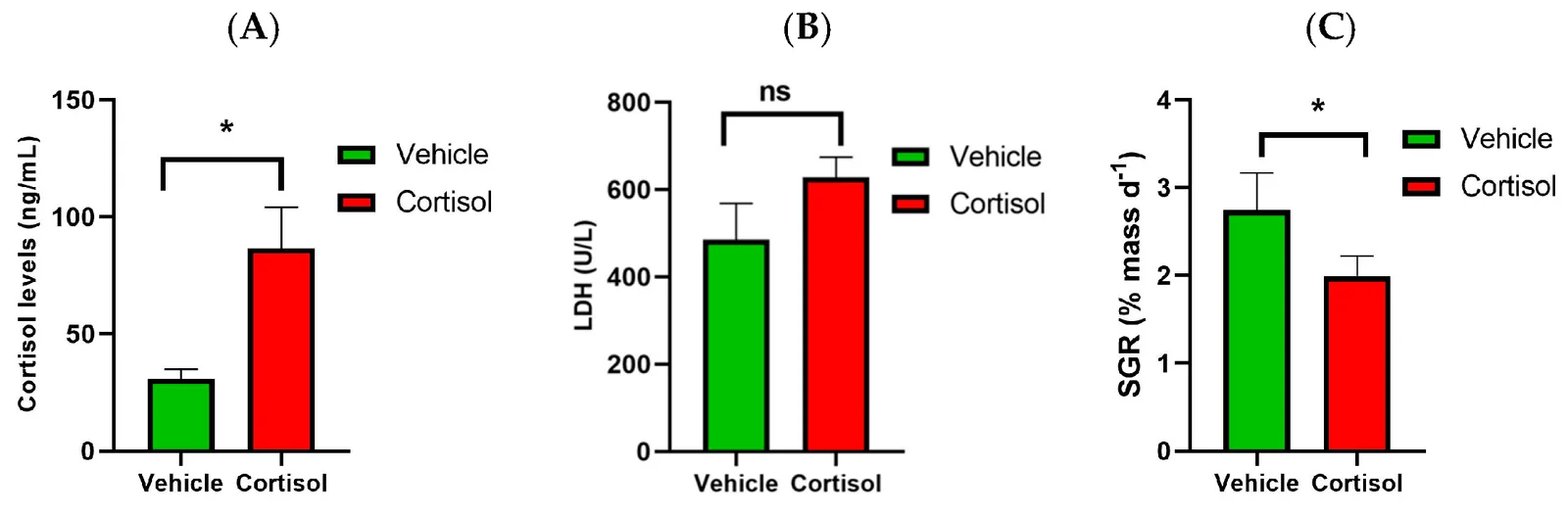
Effects of sustained cortisol elevation on plasma cortisol, LDH activity, and growth performance in rainbow trout. Plasma cortisol levels (**A**), plasma lactate dehydrogenase (LDH) activity (**B**), and specific growth rate (SGR) (**C**) were assessed in rainbow trout treated with vehicle or cortisol for 14 days. Results are expressed as means ± standard error. Differences between Vehicle and Cortisol groups are indicated as * *p*-value < 0.05; ns, not significant.

**Figure 2:**
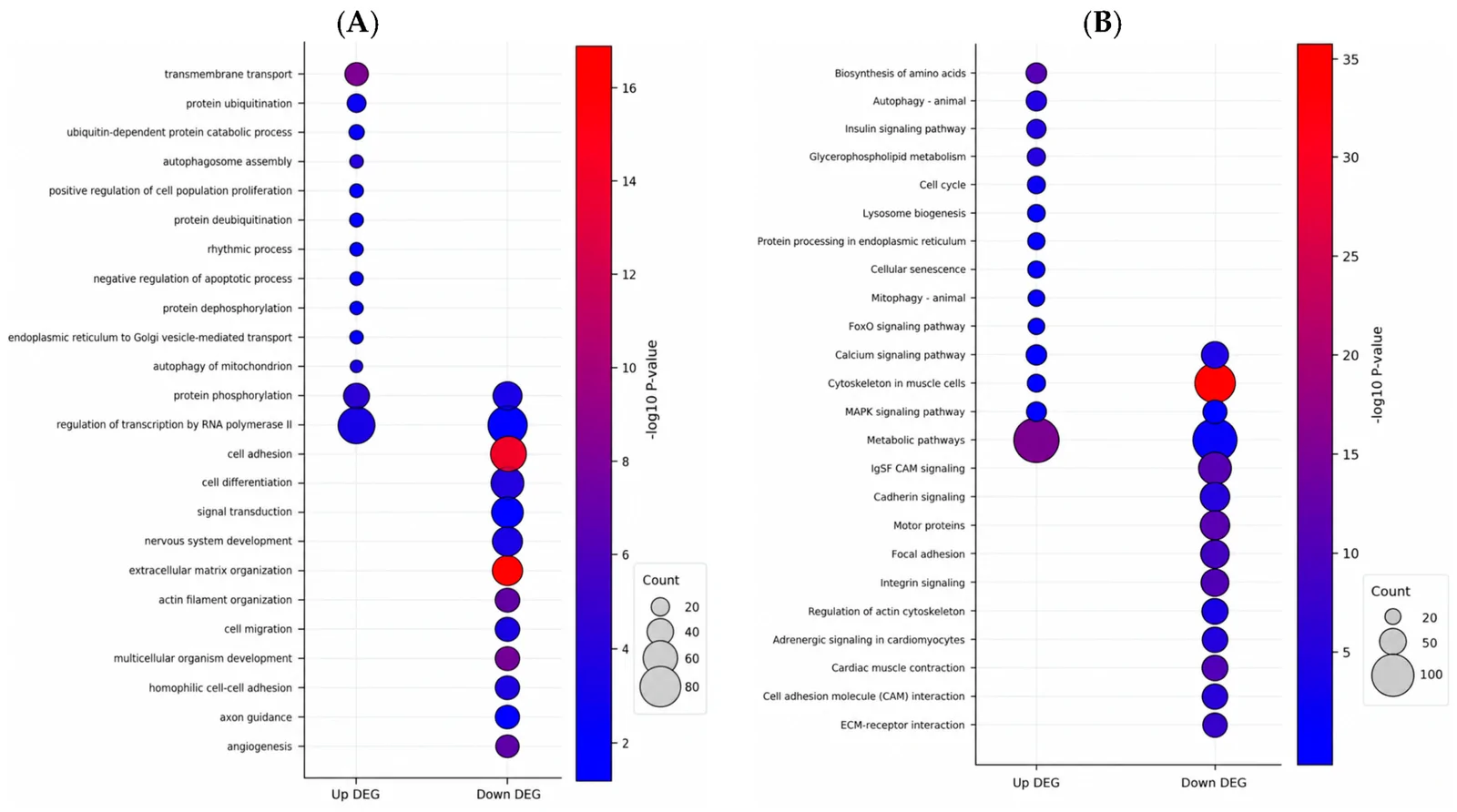
Functional enrichment analysis of differentially expressed genes (DEGs). (**A**) Gene Ontology (GO) enrichment analysis of Biological Process terms and (**B**) Kyoto Encyclopedia of Genes and Genomes (KEGG) pathway enrichment analysis for upregulated (Up DEG) and downregulated (Down DEG) genes. Bubble size represents the number of DEGs associated with each term or pathway (Count), while the color scale indicates the statistical significance of enrichment, expressed as −log10(*p*-value).

**Figure 3:**
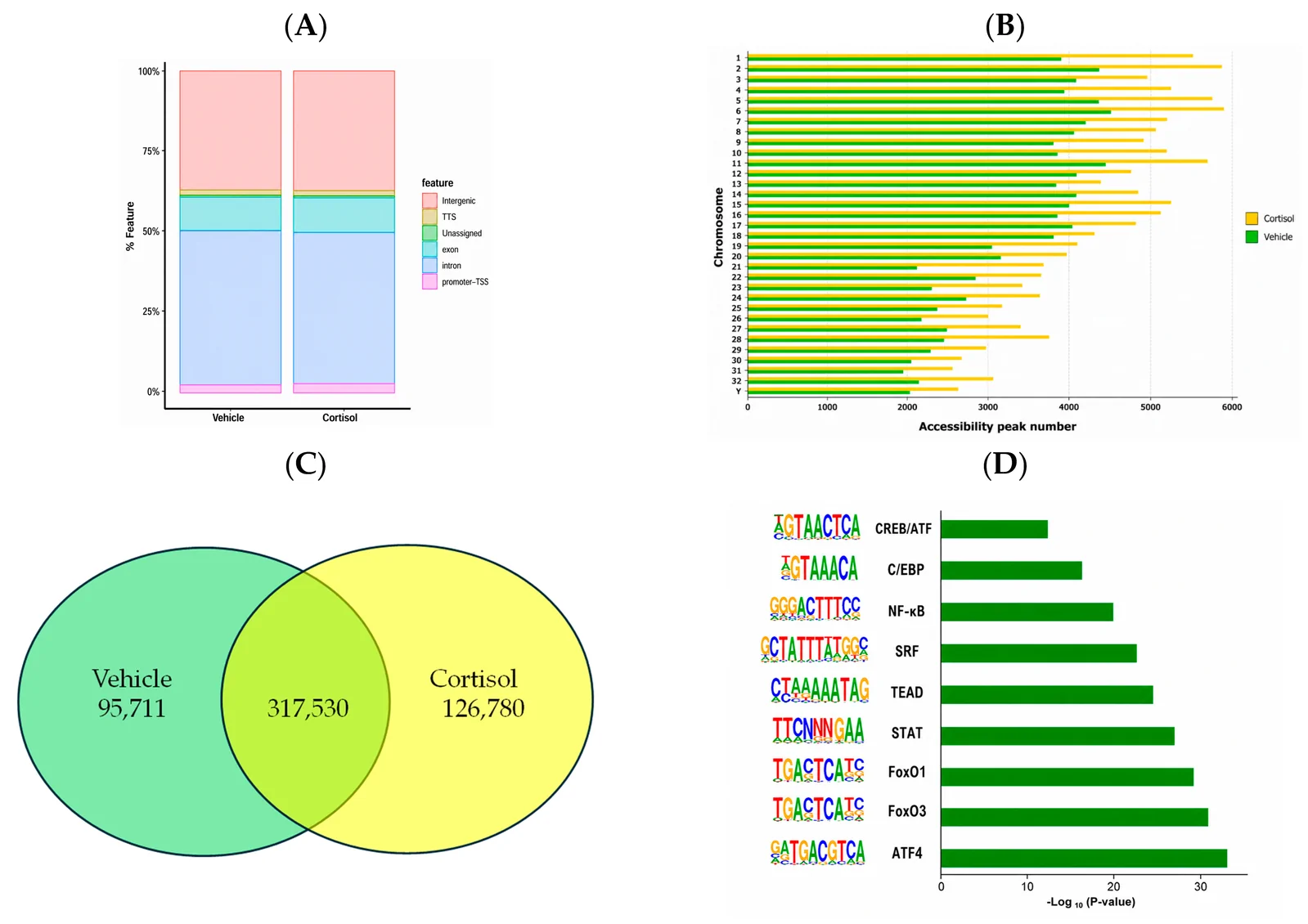
Chromatin accessibility and transcription factor motif enrichment in rainbow trout skeletal muscle following chronic cortisol exposure. (**A**) Genomic distribution of accessible chromatin peaks across promoter–TSS, intergenic, exon, intron, transcription termination site (TTS), and unassigned regions in vehicle- and cortisol-treated fish. (**B**) Chromosomal distribution of accessible peaks in the vehicle and cortisol groups. (**C**) Venn diagram showing shared and treatment-specific accessible chromatin regions. (**D**) Enrichment of transcription factor binding motifs associated with differentially accessible genes (DAGs). Motif enrichment significance is expressed as −log_10_(*p*-value).

**Figure 4:**
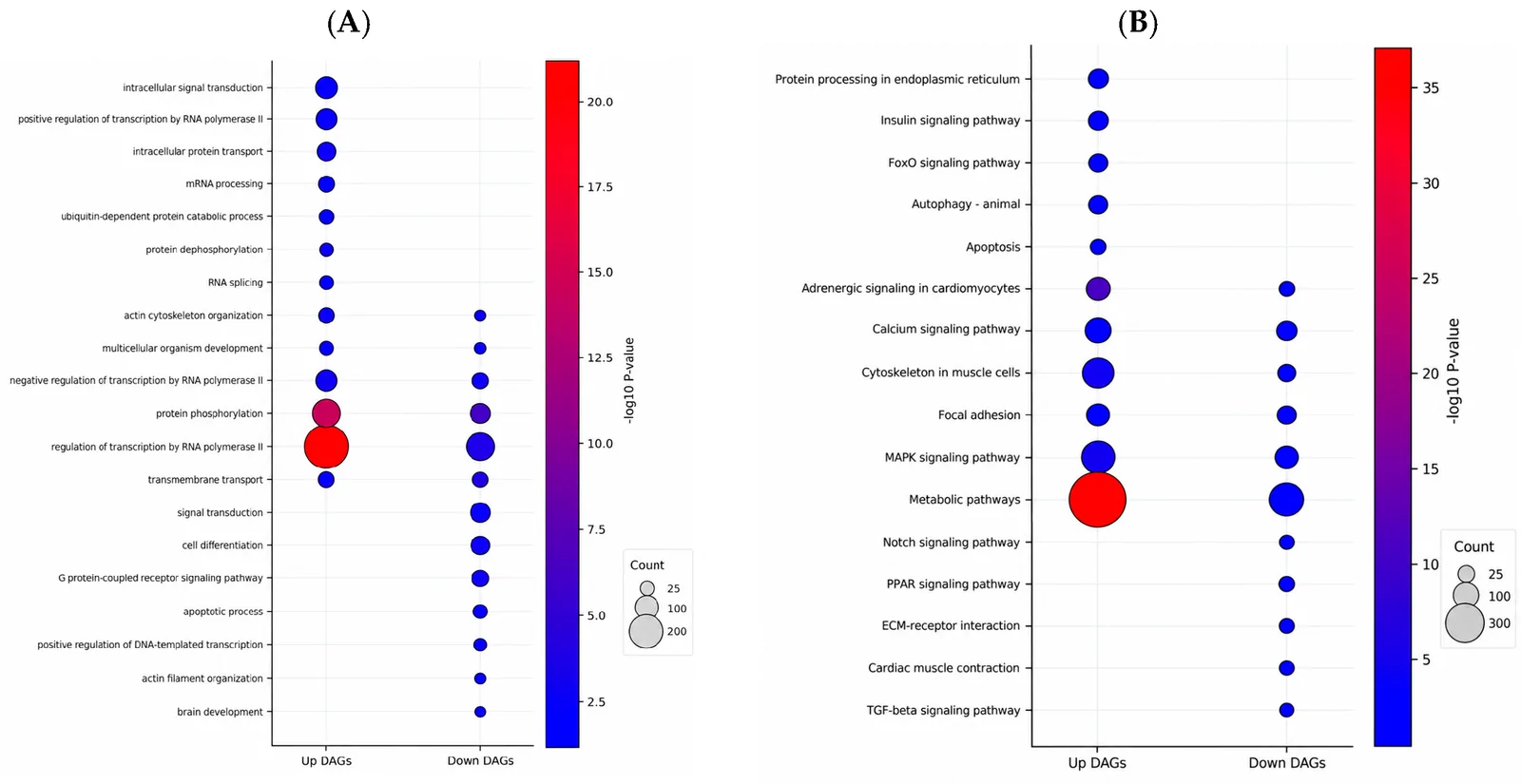
Functional enrichment analysis of differentially accessed genes (DAGs). (**A**) Gene Ontology (GO) enrichment analysis of Biological Process terms and (**B**) Kyoto Encyclopedia of Genes and Genomes (KEGG) pathway enrichment analysis for upregulated (Up DAGs) and downregulated (Down DAGs) accessed genes. Bubble size represents the number of DAGs associated with each term or pathway (Count), while the color scale indicates the statistical significance of enrichment, expressed as −log_10_(*p*-value).

**Figure 5:**
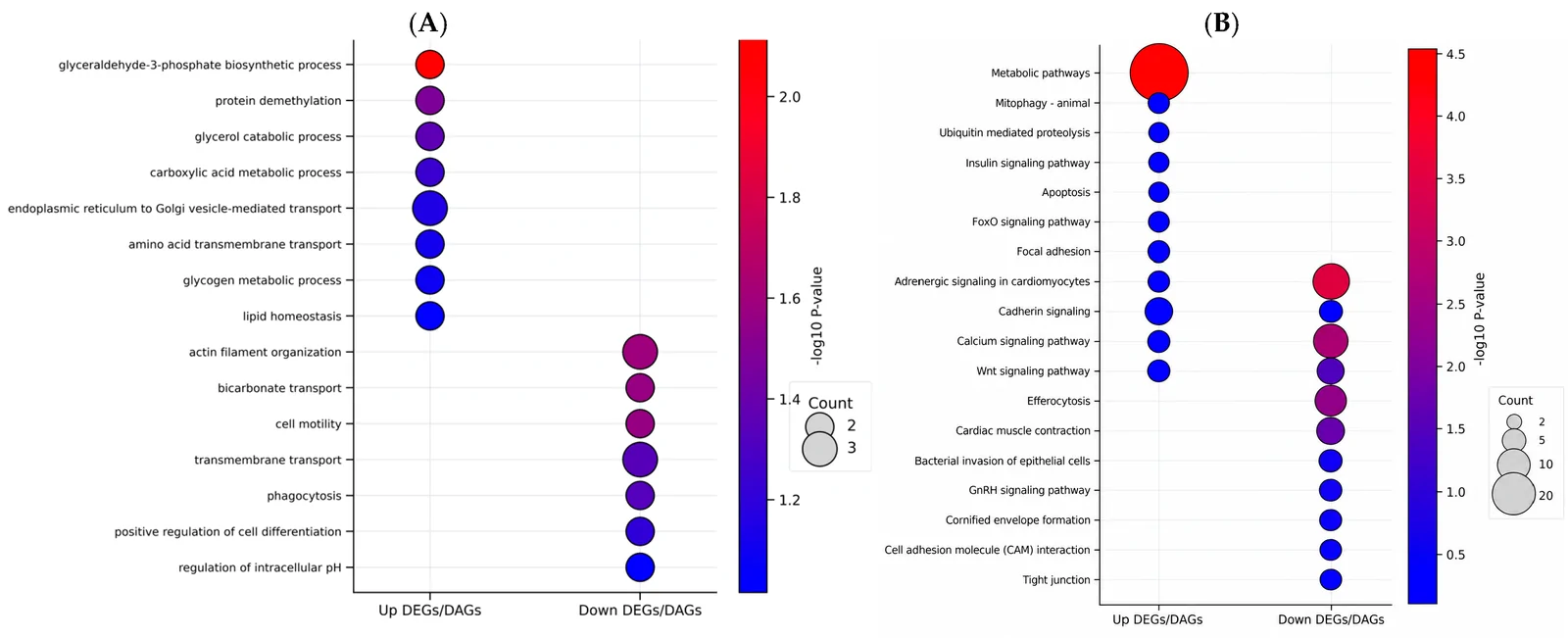
Integrative analysis of ATAC-seq and RNA-seq: (**A**) Gene Ontology (GO) enrichment analysis of Biological Process terms and (**B**) Kyoto Encyclopedia of Genes and Genomes (KEGG) pathway enrichment analysis for upregulated (Up DEGs/DAGs) and downregulated (Down DEGs/DAGs) expressed and accessed genes. Bubble size represents the number of DEGs/DAGs associated with each term or pathway (Count), while the color scale indicates the statistical significance of enrichment, expressed as −log10(*p*-value).

**Figure 6:**
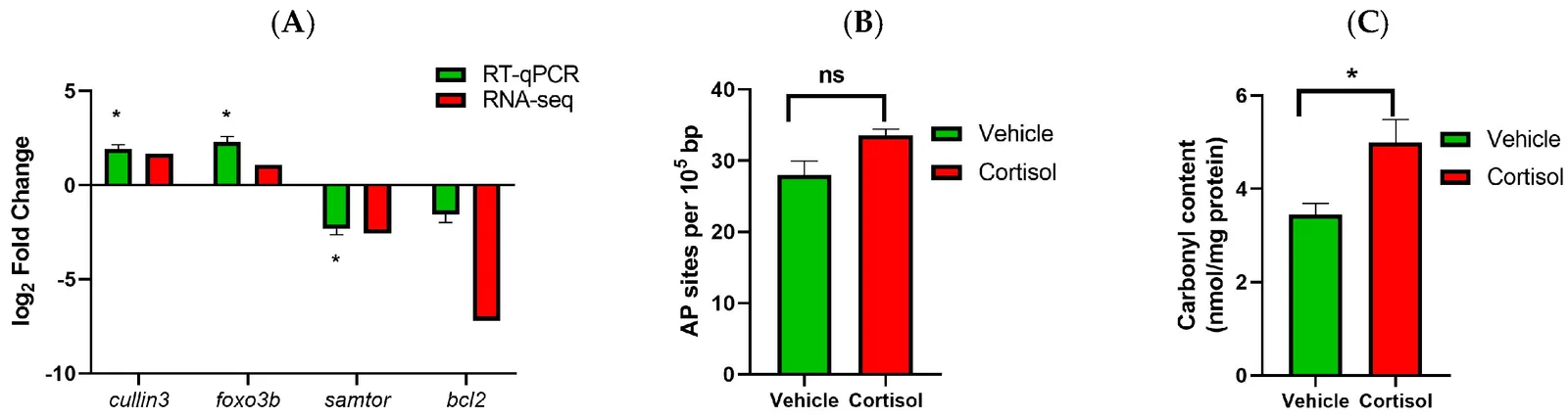
Validation of RNA-seq results and assessment of oxidative damage in rainbow trout skeletal muscle following chronic cortisol exposure. (**A**) RT-qPCR validation of selected genes showing concordant changes in chromatin accessibility and transcript abundance (*cullin-3*, *foxo3b*, *samtor*, and *bcl2*). RNA-seq and RT-qPCR results are expressed as log_2_ fold change relative to the vehicle group. RT-qPCR expression levels were normalized using *fau* and *actβ* as reference genes. (**B**) DNA oxidative damage assessed by quantification of apurinic/apyrimidinic (AP) sites. (**C**) Protein oxidative damage assessed by protein carbonyl content. Data are presented as mean ± standard error (n = 6 per treatment). Asterisks indicate significant differences between vehicle- and cortisol-treated groups (*p*-value < 0.05); ns, not significant.

## Data Availability

The data presented in this study are openly available in the Sequence Read Archive (SRA) under the following accession numbers: Bioproject PRJNA1516532 (SRR40436855, SRR40436856, SRR40436853, SRR40436851, SRR40436852, SRR40436854, SRR40296274, SRR40296275, SRR40296273, SRR40296271, SRR40296272, SRR40296270).

## Supplementary Materials

The following supporting information can be downloaded at https://www.mdpi.com/article/10.3390/ijms27198482/s1.

## Abbreviations

The following abbreviations are used in this manuscript:

GR	Glucocorticoid receptor
DAGs	Differentially accessed genes
DEGs	Differentially expressed genes
ATAC-seq	Assay for Transposase-Accessible Chromatin using sequencing
RNA-seq	RNA sequencing
SGR	Specific growth rate
