# Supplementary material for: Chronic Cortisol Exposure Reshapes Chromatin Accessibility and Gene Expression: Implications for Growth and Welfare in Rainbow Trout (Oncorhynchus mykiss)
Source: Int J Mol Sci. 2026 Sep 23;27(19):8482. doi: 10.3390/ijms27198482 (PMC13664911; doi:10.3390/ijms27198482)
Supplement: Supplementary file 1 [file ijms-27-08482-s001.zip › supplementary Figure S1.pdf]

Supplementary Figure S1

A)

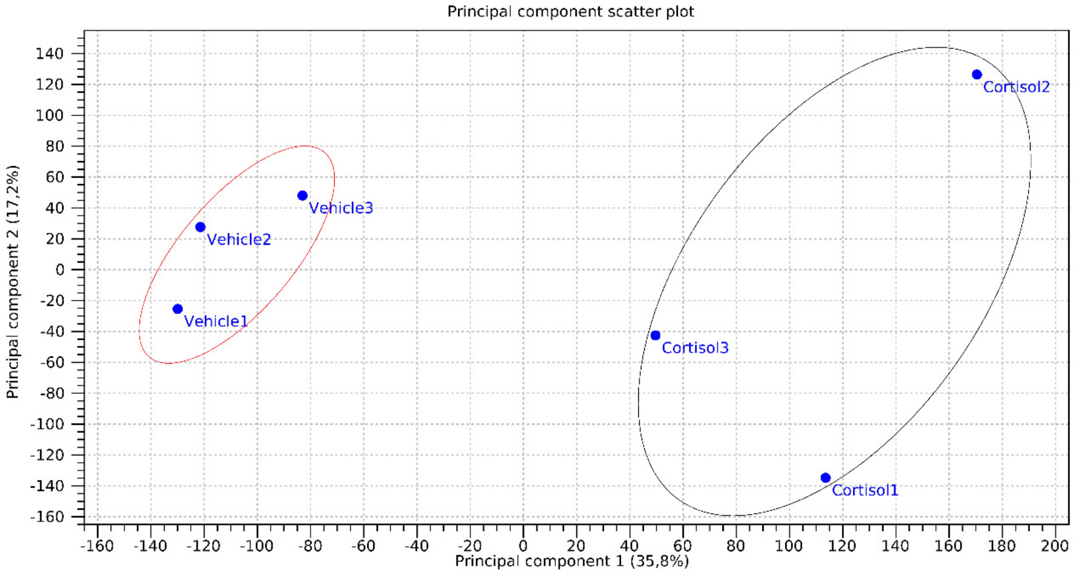

B)

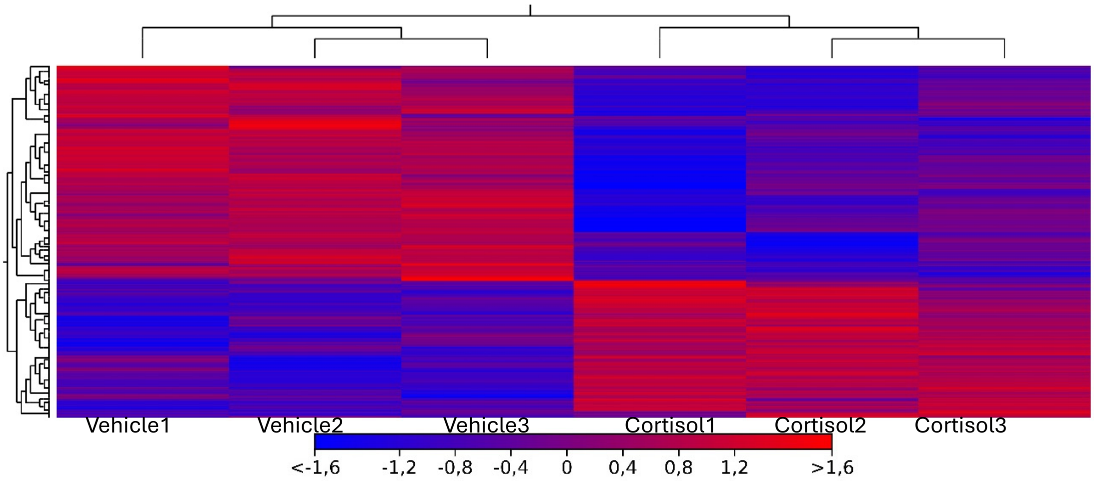

Figure S1. Transcriptomic response of rainbow trout skeletal muscle following chronic cortisol exposure. (A) Principal component analysis (PCA) of RNA-seq data from skeletal muscle of vehicle- and cortisol-treated rainbow trout after 14 days of treatment. Each point represents an individual biological replicate. (B) Heatmap showing the expression profiles of differentially expressed genes (DEGs) across vehicle and cortisol-treated samples. Gene expression values are displayed as normalized expression levels, illustrating the distinct transcriptional profiles between experimental groups.
