# Supplementary material for: Chronic Cortisol Exposure Reshapes Chromatin Accessibility and Gene Expression: Implications for Growth and Welfare in Rainbow Trout (Oncorhynchus mykiss)
Source: Int J Mol Sci. 2026 Sep 23;27(19):8482. doi: 10.3390/ijms27198482 (PMC13664911; doi:10.3390/ijms27198482)
Supplement: Supplementary file 1 [file ijms-27-08482-s001.zip › supplementary Figure S2.pdf]

Supplementary Figure S2

A)

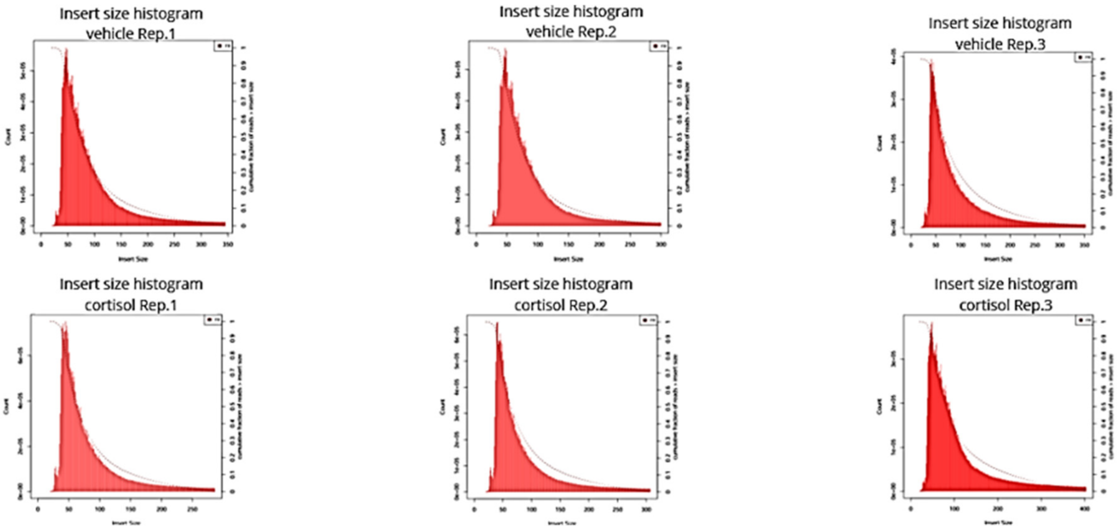

B)

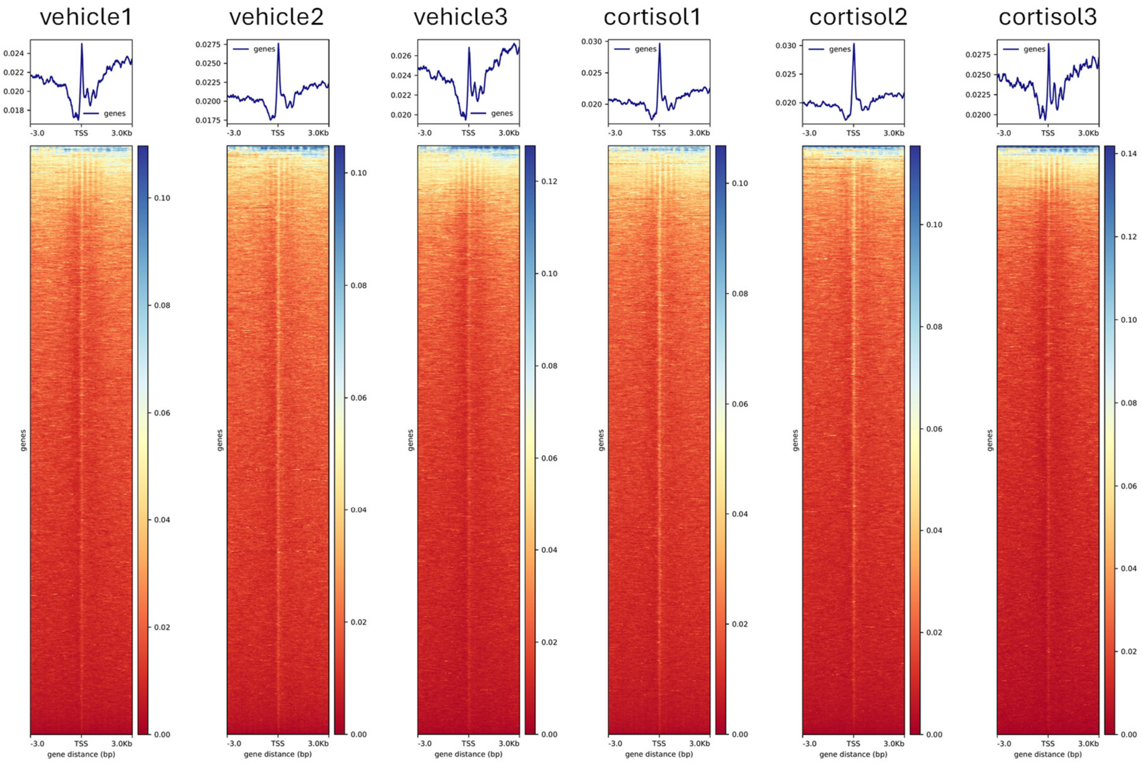

Figure S2. Quality assessment and genome-wide chromatin accessibility profiles of ATAC-seq libraries from rainbow trout skeletal muscle. (A) Fragment size distribution of ATAC-seq libraries from vehicle- and cortisol-treated fish, showing enrichment of nucleosome-free fragments and the characteristic periodic distribution of

nucleosome-associated fragments. (B) Heatmap showing ATAC-seq signal intensity across accessible chromatin regions in vehicle- and cortisol-treated samples, illustrating genome-wide chromatin accessibility patterns following chronic cortisol exposure.
