# Supplementary material for: Chronic Cortisol Exposure Reshapes Chromatin Accessibility and Gene Expression: Implications for Growth and Welfare in Rainbow Trout (Oncorhynchus mykiss)
Source: Int J Mol Sci. 2026 Sep 23;27(19):8482. doi: 10.3390/ijms27198482 (PMC13664911; doi:10.3390/ijms27198482)
Supplement: Supplementary file 1 [file ijms-27-08482-s001.zip › supplementary Figure S3.pdf]

Supplementary Figure S3

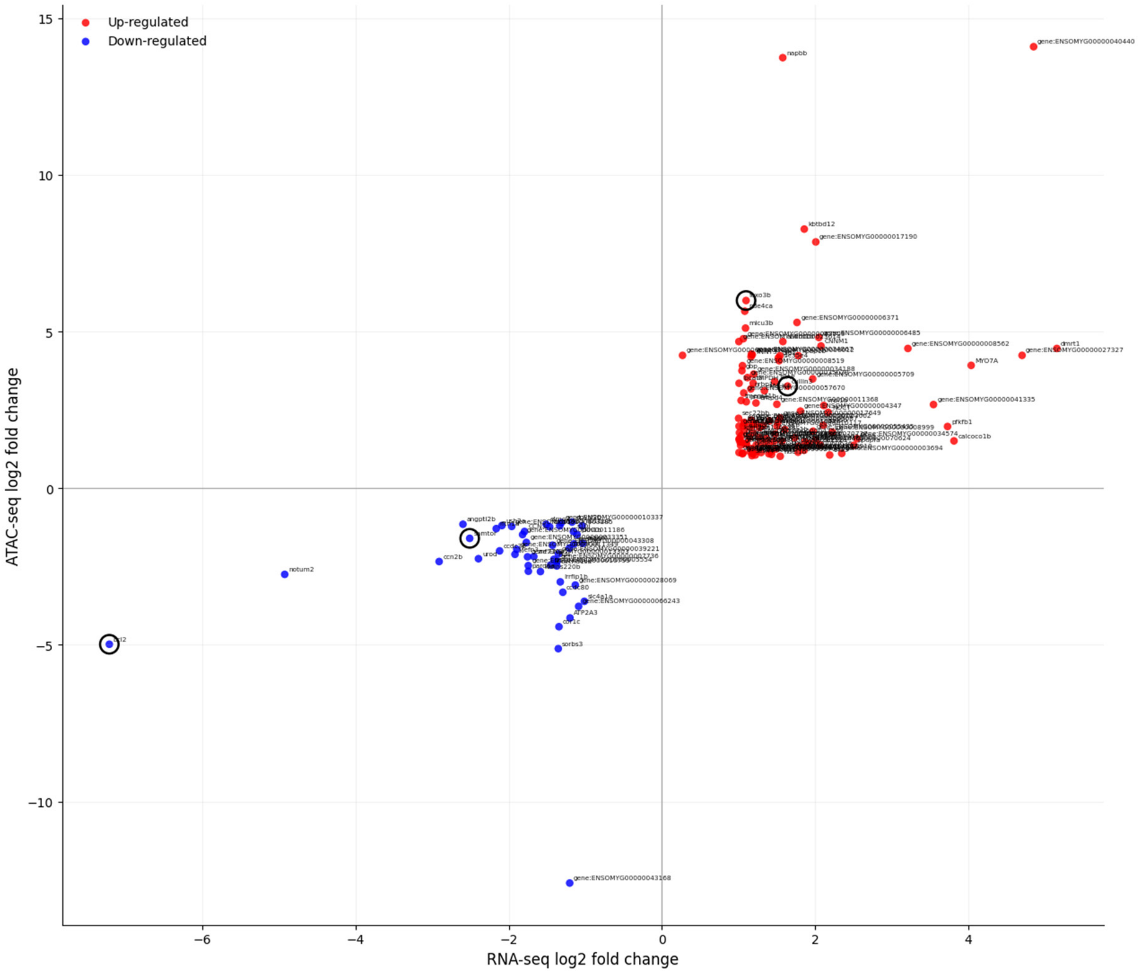

Figure S3. Integrated analysis of chromatin accessibility and gene expression in skeletal muscle following chronic cortisol exposure. Scatter plot showing the relationship between RNA-seq log2 fold change (x-axis) and ATAC-seq log2 fold change (y-axis). Each point represents a gene showing concordant changes in chromatin accessibility and transcript abundance. Upregulated genes are shown in red and downregulated genes in blue. The genes *foxo3b*, *cullin*-3, *bcl2*, and *samtor*, selected for RT-qPCR validation, are highlighted with black circles.
